# Bietti crystalline dystrophy in Türkiye: A genetic crossroads between Asia and Europe

**DOI:** 10.1007/s00417-026-07245-w

**Published:** 2026-04-20

**Authors:** Fulya Yaylacıoğlu Tuncay, Neslihan Sinim Kahraman, Gülsüm Kayhan, Zekeriya Çetinkaya, Munis Dündar, Ayşe Öner

**Affiliations:** 1 Department of Medical Biology, Gulhane Medical Faculty, University of Health Sciences, Ankara, Türkiye; 2 Department of Ophthalmology, Acibadem Hospital, İstanbul, Türkiye; 3 Department of Medical Genetics, Faculty of Medicine, Gazi University, Ankara, Türkiye; 4 Department of Ophthalmology, Kayseri State Hospital, Kayseri, Türkiye; 5 Department of Medical Genetics, Faculty of Medicine, Erciyes University, Kayseri, Türkiye

**Keywords:** Bietti crystalline dystrophy, CYP4V2, c.802–8_810del17insGC, Next-generation sequencing

## Abstract

**Purpose:**

To analyse the genotype and phenotype characteristics of Turkish patients clinically diagnosed with Bietti Crystalline Dystrophy (BCD).

**Methods:**

The clinical findings and the genetic testing results of patients diagnosed with BCD were analyzed retrospectively.

**Results:**

Thirty-two patients from 26 families were included in the study. Parental consanguinity was reported in 14 patients (43.7%). All patients carried biallelic pathogenic or likely pathogenic variants in *CYP4V2*. In total, 12 different *CYP4V2* variants were identified, three of which were novel: c.802-1G > A, c.802–2 A > C, and c.464 A > C (p.His155Pro). The most common variants were c.1198 C > T (p.Arg400Cys), with an allelic frequency of 23.4% (15/64), and c.802–8_810del17insGC, with an allelic frequency of 18.8% (12/64). Clinical features, including age of onset, visual acuity, disease stage, and optical coherence tomography (OCT) findings, did not differ significantly between genotype groups (*p* > 0.05). Interocular asymmetry in visual acuity and OCT parameters, as well as variability of clinical findings between siblings and among unrelated patients carrying the same *CYP4V2* variants, were observed in the cohort.

**Conclusion:**

This study, one of the largest European cohorts and the first comprehensive series from Türkiye, broadens the mutational spectrum of *CYP4V2* with three novel variants and highlights the marked phenotypic variability of BCD, even within families. The unexpectedly high frequency of c.802–8_810del17insGC in the Turkish population, previously reported mainly in East Asia, provides new insights into the geographic distribution of this variant.

**Supplementary Information:**

The online version contains supplementary material available at https://doi.org/10.1007/s00417-026-07245-w.

## Key messages

*****What is known***:**

- Most BCD phenotype–genotype data are from East Asia.
- The common *CYP4V2* variant c.802–8_810del17insGC has been reported almost exclusively in East Asian patients.

*****What is new***:**

- Identification of three novel *CYP4V2* variants.
- Unexpectedly high frequency of c.802–8_810del17insGC in the Turkish population.
- Underlines the need for population-specific genotype–phenotype studies to improve patient management.
- Suggests Türkiye’s geographic and historical context may reveal further unexpected variant distributions in genetic diseases.

**Supplementary Information:**

The online version contains supplementary material available at https://doi.org/10.1007/s00417-026-07245-w.

## Introduction

Bietti crystalline corneoretinal dystrophy (BCD; MIM210370) is a rare, autosomal recessive inherited eye disease. Up to now, patients have been reported from several countries; however, the prevalence of BCD is higher in East Asian countries, including China and Japan [1]. The disease is characterized by multiple small yellow-white crystalline deposits mainly in the retina associated with atrophy of the retinal pigment epithelium (RPE), choroid, and the photoreceptors [2]. In a variable percentage of patients, crystalline deposits can also be detected in perilimbal subepithelial cornea, other corneal layers, and lens [3–5]. Furthermore, lymphocytes and skin fibroblasts of patients were reported to have similar deposits; however, patients experience only visual symptoms, including decreased vision, night blindness, and visual field defects [1]. BCD is usually diagnosed in the second or third decade, and due to the progressive nature of the disease, legal blindness can develop by the fifth or sixth decade of life [1, 2].

Clinical diagnosis and staging of the disease can be based on fundus features [6]. Since it is known that BCD is caused by homozygous or compound heterozygous mutations in the *CYP4V2* gene, genetic testing can be used to confirm the clinical diagnosis or as a primary diagnostic test in challenging cases [7]. The *CYP4V2* gene is located in 4q35.1-q35.2 and encodes a 525 amino acid protein, a member of the cytochrome P450 family, and converts fatty-acid precursors into n-3 polyunsaturated fatty acids [8]. Defective or loss of function of the protein causes lipid metabolism problems leading to cholesterol accumulation in the retina, cornea, lymphocytes, and fibroblasts [8, 9].

More than 120 mutations in the *CYP4V2* gene have been reported in BCD patients on the Human Genome Mutation Database (HGMD) website (https://www.hgmd.cf.ac.uk, accessed on 5 May 2025). Most genotype-phenotype association studies have focused on patients from East Asia, where the most frequent mutation identified is c.802–8_810del17insGC, a splicing mutation that accounts for more than half of the mutant alleles observed in East Asian patients [10, 11]. Smaller cohorts from other regions including Italy, Spain, the UK, India, Lebanon, Iran, and Brazil have also contributed to the mutation spectrum of the *CYP4V2* gene in BCD patients [12–21]. To date, reports of BCD patients from Türkiye are limited, with only one study describing eight patients with genetic data [22–24]. Therefore, this study aimed to provide the first comprehensive genotype–phenotype characterization of Turkish BCD patients.

## Methods

### Participants and clinical evaluation

Patients who were clinically diagnosed with BCD and followed at the ophthalmology clinic of Kayseri and Taksim Acibadem Hospital between January 2018 and December 2024 were included in this retrospective study. All patients underwent a comprehensive medical anamnesis and ophthalmological examination. Following clinical diagnosis, all the patients were referred to a medical geneticist for genetic counselling and genetic testing. Medical records, images, and genetic test reports of patients were reviewed retrospectively. Age at examination (AE), sex, age of onset (AO), family history, and parental consanguinity were recorded. Best corrected visual acuity (BCVA), intraocular pressure, biomicroscopy, fundus examination, color fundus photography (CFP), optical coherence tomography (OCT), visual field (VF) testing using the Humphrey 30 − 2 program (Carl Zeiss Meditec AG, Germany) were performed in all patients. Central macular thickness (CMT) and subfoveal choroidal thickness (SCT) were assessed in OCT images [19]. Two different graders measured CMT and SCT, and the values were calculated as an average. A horizontal OCT slice through the foveal centre was used for measurement. CMT was measured manually as the distance from the inner limiting membrane at the centre of the fovea to the RPE. Electrophysiological testing with multifocal electroretinogram (ERG) and full-field ERG (Metrovision, France) according to international standards was performed on 11 patients in the study [25]. The diagnosis of BCD was based on patients’ symptoms and ophthalmoscopic findings, and the stage classification of Yuzawa et al. was used [6]. Changes in BCVA per year were calculated using the follow-up duration and the LogMAR BCVA values from the first and last examinations of patients for whom sufficient data were available. Interocular asymmetry was assessed for both functional and structural parameters. For BCVA, asymmetry was defined as a difference greater than two lines between the two eyes [26]. For OCT and VF parameters, asymmetry was evaluated using an asymmetry index (AI), calculated as the absolute difference between the two eyes divided by their mean. AI values exceeding the 95th percentile of the reference distribution were considered indicative of significant asymmetry [27].

### Genetic analysis

QIAamp^®^ DNA Mini Kit (QIAGEN, Ankara, Türkiye) was used for DNA extraction from the peripheral venous blood. The targeted gene analysis (*CYP4V2*-Twist custom kit) was used for the library and exome enrichment. The manufacturer’s protocols were used for all procedures. Paired-end sequencing was performed on an MGI Tech, DNBSEQ-G400 system with a read length of 150 × 2. The *CYP4V2 *gene had an average read depth of 80X, with 100% coverage of exons and exon-intron boundaries at 50X reads. The Massive analyzer (Massive Bioinformatics, Türkiye) or the Ilyome software (Ilyome, Türkiye) was used to analyze sequencing data. In these platforms, raw fastq files were briefly converted to binary alignment and map (bam) by aligning to the human reference genome (GRCh38), and finally variant call format (VCF) files. Using the same platform, variants were filtered according to the minor allele frequency (MAF) < 0.01% based on gnomAD v4.1.0 and in-house frequency data. After filtering, all remaining variants were classified according to the American College of Medical Genetics and the Association for Clinical Genomic Science standards [28, 29].

### Statistical analysis

The study data were analyzed using SPSS for Windows version 21.0 (IBM Corp., Armonk, NY, USA). Descriptive statistics (mean, range) were used to evaluate the data. Pearson chi-square and Fisher exact tests were used to compare categorical values, and Mann-Whitney U test was used to compare continuous values. A p-value less than 0.05 was considered as significant.

## Results

### Demographic and clinical findings

Thirty-two BCD patients from 26 families were included in this study. The demographic, clinical and genetic findings of the patients are summarized in Tables 1 and 2 and presented in detail in Supplementary Table 1. Eighteen patients were female, and 14 were male. The mean AE of patients was 37.3 years, ranging from 12 to 61, and the mean AO of patients was 21.9 years, ranging from 10 to 42.

**Table 1:** Demographic and clinical characteristics of Turkish BCD patients

No	Sex	Province	AE	AO	BCVA		Stage	OCT				VF (MD)		BCVA change/year		Follow-up time (year)
					OD	OS		ORT	ME	CMT*	SCT*	OD	OS	OD	OS	
P1	F	Kayseri	49	30	0.2	0.3	2	OU	N	181	69	-28.23	-27.76	0	0	3
P2	F	Kayseri	40	25	0.05	0.01	2	N	OU	146	45	-17.26	-21.18	0.1	0.14	5
P3	F	Elazığ	49	20	0.01	0.01	3	N	N	66	37	-31.33	-31.8	0.05	0.05	6
P4	M	Elazığ	36	25	0.3	0.6	2	OU	N	155	225	-32.2	-32.66	0	0.09	2
P5	M	Elazığ	34	10	0.7	0.7	2	OU	N	192	268	-5.61	-10.7	NA	NA	NA
P6	F	Kayseri	61	36	0.01	0.01	3	N	N	73	55	-33.1	-33.45	0.08	0.08	4
P7	M	İstanbul	36	20	0.01	0.01	3	N	N	105	75	-32.4	-31.86	0.14	0.14	5
P8^a^	F	Kayseri	31	22	0.001	0.001	3	N	N	82	42	-33.78	-33.82	0.2	0.2	5
P9^a^	F	Ordu	24	18	0.3	0.2	1	OU	N	172	106	-21.65	-18.9	0	0	4
P10	F	Konya	58	40	0.8	0.8	1	N	N	185	198	-14.6	-16.8	0.01	0.01	7
P11	F	Malatya	35	20	0.2	0.16	2	OU	N	111	168	-22.95	-25.43	0	0	5
P12	F	Niğde	39	20	0.01	0.05	3	OU	N	33	30	-26.97	-23.11	0.14	0	5
P13	M	İstanbul	28	17	0.1	0.01	3	OU	N	156	87	-31.54	-27.06	0.04	0.14	5
P14	M	Kocaeli	42	25	0.5	0.1	3	OU	N	75	130	-32.7	-33.52	0.02	0.17	4
P15	F	İstanbul	28	15	0.4	0.001	3	N	N	35	45	-31.68	-33.76	0	0.08	6
P16	M	İstanbul	50	35	0.3	0.3	2	N	N	90	44	-31.07	-26.79	0	0	3
P17	M	Giresun	38	15	0.05	0.3	2	N	N	148	58	-28.23	-27.76	0	0	4
P18^b^	F	İstanbul	37	19	0.001	0.001	3	N	N	45	45	-33.76	-33.81	0	0	2
P19^b^	F	İstanbul	36	25	0.01	0.05	3	N	N	70	55	-27.26	-32.71	0	0	3
P20^c^	F	İstanbul	14	10	0.8	0.8	1	OU	OU	198	235	-20.49	-21.74	0.05	0.05	6
P21^c^	M	İstanbul	12	11	1.0	1.0	1	N	N	222	325	-3.5	-4.2	NA	NA	NA
P22	F	Kocaeli	45	22	0.001	0.001	3	N	N	25	10	-32.18	-32.79	0	0	4
P23^d^	F	Kayseri	52	25	0.3	0.05	3	OU	N	47	24	-31.64	-32.96	0	0.5	2
P24^d^	M	Kayseri	43	18	0.1	0.1	3	N	N	99	52	-31.53	-31.96	0	0	2
P25^e^	F	Antalya	39	25	0.05	0.05	3	OU	N	182	72	-31.77	-31.46	0.08	0.08	6
P26^e^	M	Antalya	42	42	0.6	0.7	2	OU	N	135	91	-13.19	-19.1	0	0	2
P27	F	Bartın	29	13	0.001	0.001	3	N	N	176	55	-33.03	-33.91	0.21	0.21	6
P28	M	Ordu	47	20	0.05	0.05	3	N	N	146	58	-32.86	-33.34	0	0	3
P29	M	Mersin	32	22	1.0	1.0	2	N	N	263	156	-31.39	-29.67	0	0	3
P30	F	Aksaray	32	20	0.5	0.5	2	N	N	87	216	-7.53	-10.86	NA	NA	NA
P31^f^	M	Aksaray	23	15	0.05	0.8	2	N	N	133	40	-19.32	-20.3	0	0,02	5
P32^f^	M	Aksaray	33	20	0.8	0.8	2	OU	N	156	124	-19.55	-17.12	0	0	5

* denotes thinnest CMT and SCT values for the patient; a, b, c, d, e, f denotes sibling groups

*P* patient, *M* male, *F* female, *AO* age of onset, *AE* age at exam, *BCVA* best corrected visual acuity in decimal, *BCVA change/year* best corrected visual acuity changes per year in LogMAR, *OD* right eye, *OS* left eye, *ORT* outer retinal tubulation, *OU* both eyes, *N* not detected, *ME* macular edema, *OCT* optic coherance tomography, *CMT* central macular thickness in µm, *SCT* subfoveal choroidal thickness in µm, *VF* visual field, *MD* mean deviation values in minus dB, *NA* not available

**Table 2:** Genetic characteristics of Turkish BCD patients

No	Variant		Number of LOF variants	Zygosity	Parental Consanguinity
	Allele 1	Allele 2			
P1	c.252 C > A, p.Tyr84Ter	c.1198 C > T, p.Arg400Cys	1	Compound heterozygous	no
P2	c.252 C > A, p.Tyr84Ter	c.1198 C > T, p.Arg400Cys	1	Compound heterozygous	no
P3	c.252 C > A, p.Tyr84Ter	c.1198 C > T, p.Arg400Cys	1	Compound heterozygous	no
P4	c.252 C > A, p.Tyr84Ter	c.252 C > A, p.Tyr84Ter	2	Homozygous	yes
P5	c.252 C > A, p.Tyr84Ter	c.252 C > A, p.Tyr84Ter	2	Homozygous	no
P6	c.1168 C > T, p.Arg390Cys	c.1168 C > T, p.Arg390Cys	0	Homozygous	no
P7	c.677T > A, p.Met226Lys	c.677T > A, p.Met226Lys	0	Homozygous	no
P8^a^	c.677T > A, p.Met226Lys	c.677T > A, p.Met226Lys	0	Homozygous	yes
P9^a^	c.677T > A, p.Met226Lys	c.677T > A, p.Met226Lys	0	Homozygous	yes
P10	c.332T > C, p.Ile111Thr	c.332T > C, p.Ile111Thr	0	Homozygous	no
P11	c.332T > C, p.Ile111Thr	c.332T > C, p.Ile111Thr	0	Homozygous	yes
P12	c.332T > C, p.Ile111Thr	c.332T > C, p.Ile111Thr	0	Homozygous	yes
P13	c.332T > C, p.Ile111Thr	c.802-1G > A	1	Compound heterozygous	no
P14	c.1198 C > T, p.Arg400Cys	c.1198 C > T, p.Arg400Cys	0	Homozygous	no
P15	c.1198 C > T, p.Arg400Cys	c.1198 C > T, p.Arg400Cys	0	Homozygous	no
P16	c.1198 C > T, p.Arg400Cys	c.1198 C > T, p.Arg400Cys	0	Homozygous	no
P17	c.802-8_810de17linsGC	c.802-8_810de17linsGC	2	Homozygous	no
P18^b^	c.802-8_810de17linsGC	c.802-8_810de17linsGC	2	Homozygous	no
P19^b^	c.802-8_810de17linsGC	c.802-8_810de17linsGC	2	Homozygous	no
P20^c^	c.802-8_810de17linsGC	c.802-8_810de17linsGC	2	Homozygous	no
P21^c^	c.802-8_810de17linsGC	c.802-8_810de17linsGC	2	Homozygous	no
P22	c.802-8_810de17linsGC	c.802-8_810de17linsGC	2	Homozygous	no
P23^d^	c.253 C > T, p.Arg85Cys	c.253 C > T, p.Arg85Cys	0	Homozygous	yes
P24^d^	c.253 C > T, p.Arg85Cys	c.253 C > T, p.Arg85Cys	0	Homozygous	yes
P25^e^	c.802–2 A > C	c.802–2 A > C	2	Homozygous	yes
P26^e^	c.802–2 A > C	c.802–2 A > C	2	Homozygous	yes
P27	c.1523G > A, p.Arg508His	c.1523G > A, p.Arg508His	0	Homozygous	yes
P28	c.694 C > T, p.Arg232Ter	c.694 C > T, p.Arg232Ter	2	Homozygous	yes
P29	c.464 A > C, p.His155Pro	c.464 A > C, p.His155Pro	0	Homozygous	yes
P30	c.1198 C > T, p.Arg400Cys	c.1198 C > T, p.Arg400Cys	0	Homozygous	no
P31^f^	c.1198 C > T, p.Arg400Cys	c.1198 C > T, p.Arg400Cys	0	Homozygous	yes
P32^f^	c.1198 C > T, p.Arg400Cys	c.1198 C > T, p.Arg400Cys	0	Homozygous	yes

a, b, c, d, e, f denotes sibling groups

*LOF* Loss of function variants (splicing and nonsense variants), *P* patient

**Fig. 1:**
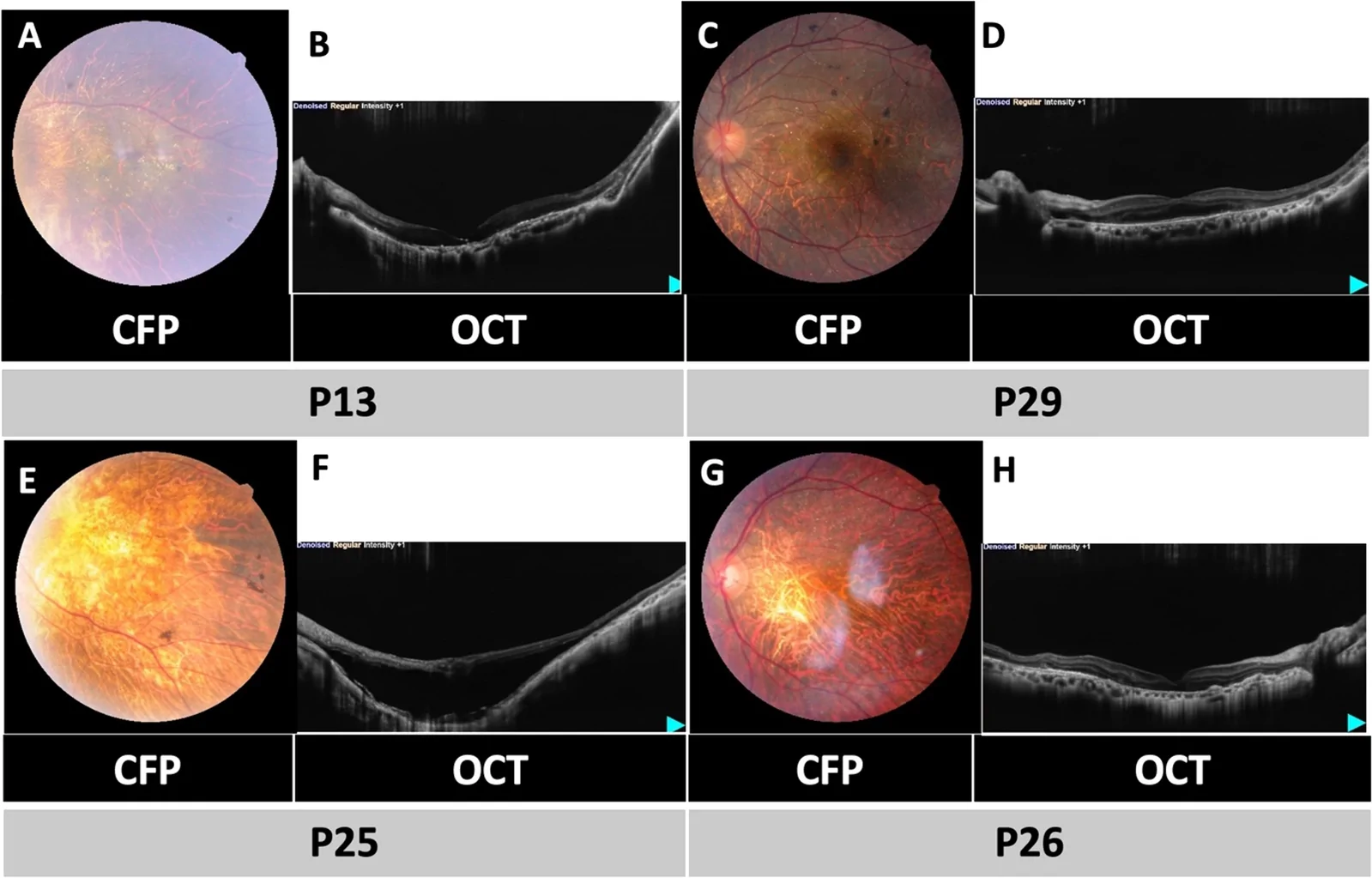
Color fundus photography (CFP) and optical coherence tomography (OCT) images of BCD patients with novel *CYP4V2* variants. A: CFP image of the left eye of P13, with the novel heterozygous splicing variant, c.802-1G > A, showing stage 3 fundus findings with widespread chorioretinal atrophy, few crystal deposits, some pigment clumping areas; B: OCT image of the left eye of P13 showing foveal and choroidal thinning; C: CFP image of the left eye of P29, with the novel homozygous missense variant, p.His155Pro, showing stage 2 fundus findings with chorioretinal atrophy extending from the posterior pole, few crystal deposits in the macula, some pigment clumping areas; D: OCT image of the left eye of P29 showing thinning in retina and choroid sparing central region; E: CFP image of the left eye of P25, with the novel homozygous splicing variant, p.His155Pro, showing stage 3 fundus findings with widespread RPE and choroidal atrophy, bone spicule pigmentation; F: OCT image of the left eye of P25 showing thinning in retina and choroid with foveoschisis; G: CFP image of the left eye of P26, older sibling of P25, showing stage 2 fundus findings with RPE atrophy, equatorial crystal deposits, some pigment clumping areas; H: OCT image of the left eye of P26 showing thinning in retina and choroid with the loss of the ISOS band

All participants self-identified as Turkish, and 15 were from central Anatolia. Parental consanguinity was reported in 14 patients (43.7%). Systemic diseases and drug use, causing the accumulation of intraretinal crystals, were excluded by medical history. The most common presenting symptoms were decreased vision and night blindness, although one patient (P29) was diagnosed incidentally during a military examination. BCVA ranged from hand motion (0.001 in decimal) to 1.0, with seven patients exhibiting asymmetry. The mean BCVA change per year in LogMAR were 0.053, ranging from 0 to 0.21 (Table 1, Supplementary Table 1). Slit-lamp examinations revealed corneal crystalline deposits at the limbus in 7 patients (21.9%). Fundus examinations revealed varying numbers of intraretinal small yellow-white crystals distributed in the posterior pole and mid-periphery in all patients (Fig. 1). Other findings included retina pigment epithelium (RPE) atrophy, chorioretinal atrophy, bone spicule pigmentation, bright macular plaques, cystoid macular edema (CME), foveoschisis, and lamellar hole. Based on fundus findings, four patients were stage 1, 12 were stage 2, and 16 were stage 3. All patients underwent OCT examination, which confirmed crystalline deposits and RPE atrophy, as well as identified outer retinal tubulation (ORT), inner segment/outer segment junction layer (ISOS) loss, CME, foveoschisis, lamellar holes, epiretinal membranes, and vitreomacular traction. ORT was present in 13 (40.6%) patients-2 in stage 1, 6 in stage 2, and 5 in stage 3. Choroidal neovascularization was not observed. The mean CMT in the thinnest eye was 117.09 𝜇m (range from 25 to 263𝜇m), and the mean SCT in the thinnest eye was 101.25 𝜇m (range from 10 to 325𝜇m). Interocular asymmetry was observed in two patients regarding CMT (P12 and P14) and SCT (P12 and P16) measurements in OCT images. Visual field testing revealed an MD value of -25.8 ± 6.4 dB in the right eye and − 26.3 ± 5.4 dB in the left, indicating significant functional loss. No interocular asymmetry was noted in VF evaluations. There was a positive correlation between BCVA and CMT/SCT (*p* < 0.001). while a significant negative correlation was found between MD and BCVA (*p* < 0.001). No correlation was observed between AO and BCVA (*p* > 0.05, Supplementary Table 2). Full field electroretinogram (ERG) revealed extremely low amplitudes in 11 patients, and multifocal ERG was available for the same group, where no correlation was found between BCVA and multifocal ERG amplitudes (*p* > 0.05).

### *CYP4V2* variants

All patients had biallelic pathogenic (P) or likely pathogenic (LP) *CYP4V2* variants. Twenty-eight patients had homozygous variants, while four had compound heterozygous variants.

A total of 12 *CYP4V2* variants were identified, predominantly missense (7/12), with additional three splicing and two nonsense variants (Table 2, Supplementary Table 3). Notably, two novel splicing variants (c.802-1G > A, c.802–2 A > C) and one missense variant (c.464 A > C; p.His155Pro) were described. None of these novel variants appeared in the GnomAD database (https://gnomad.broadinstitute.org/gene/ENSG00000145476, last accessed 5 May 2025) or the Turkish Genome Project Data Sharing Portal (https://tgd.tuseb.gov.tr/tr/gene/ENSG00000145476, last accessed on 5 May 2025), and all were classified as LP based on ACMG criteria.

The most prevalent variant was c.1198 C > T (p.Arg400Cys), found in 9 patients (3 heterozygous, 6 homozygous) from 8 unrelated families, with an allelic frequency of 23.4% (15/64). Another common variant was c.802–8_810del17insGC, present in 6 patients from 4 unrelated families, with an allele frequency of 18.7% (12/64). The variant c.252 C > A (p.Tyr84Ter) was observed in 5 unrelated patients (3 heterozygous, 2 homozygous), with a frequency of 10.9% (7/64).

**Fig. 2:**
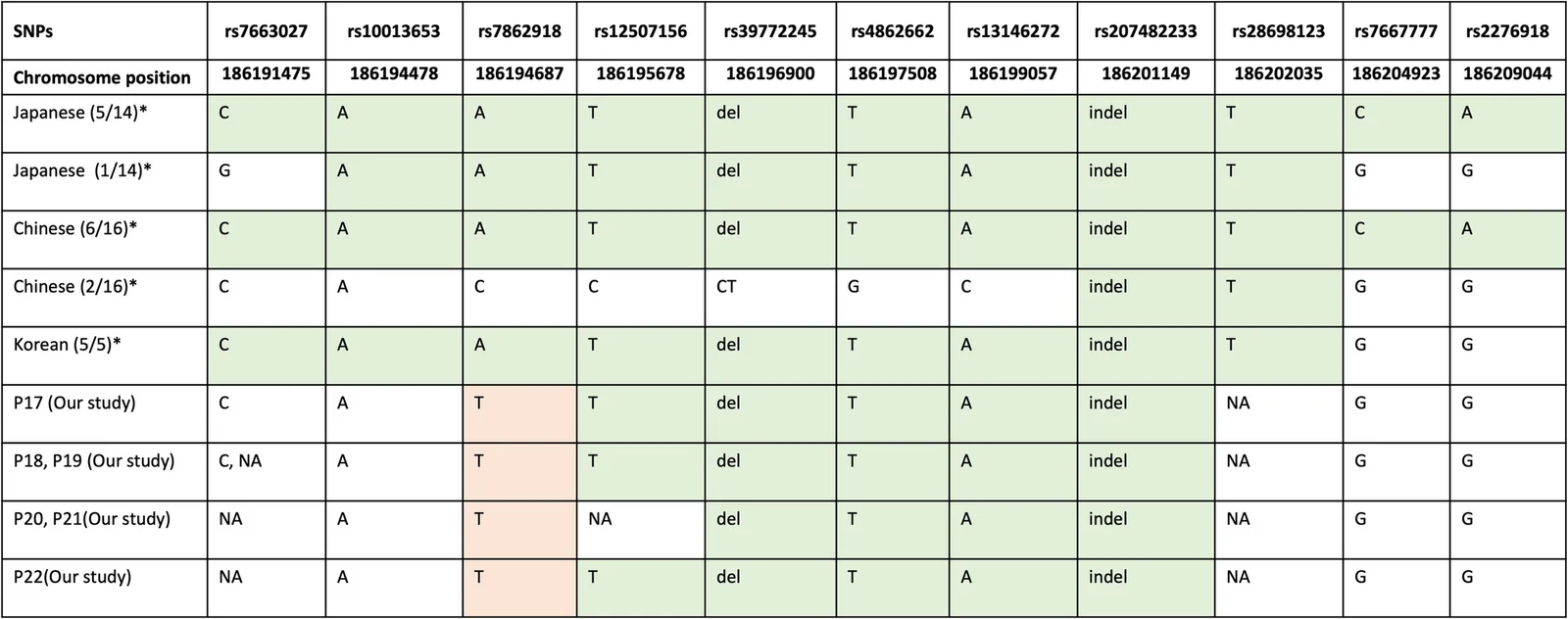
Schematic representation of the *CYP4V2* haplotype structures surrounding the pathogenic variant c.802-8_810del17insGC in previously reported East Asian populations (Jiao et al.) [11] and in our study cohort. Conserved allele blocks are shaded in green. The pink-shaded SNP (rs7862918, C > T) was observed only in our study cohort.* indicates the number of alleles carrying the shown haplotype / total number of alleles harboring the pathogenic variant in the respective populations. NA, genotype data not available

For c.802–8_810del17insGC, intragenic SNP haplotypes from our patients were aligned and compared with previously reported Chinese, Japanese, and Korean haplotypes (Fig. 2) [11]. This comparison demonstrated substantial overlap with the previously reported East Asian founder haplotype CAAT(delCT)TA(indel)TCA. Although none of the Turkish patients carried the complete East Asian founder haplotype, all individuals harboring the homozygous c.802–8_810del17insGC variant shared a conserved nine-SNP haplotype block spanning from rs10013653 to rs2276918 surrounding the mutant allele. The four SNPs immediately flanking the mutation site were identical to the previously reported Chinese founder haplotype, whereas divergence was observed at the fifth upstream marker, rs7862918, which consistently carried the T allele in Turkish patients instead of A or C alleles reported in East Asian populations. In addition, the last two SNPs (rs7667777 and rs2276918) also differed from the ancestral haplotype in all mutation carriers; however, they were identical to the Korean haplotypes. Neither the previously reported founder haplotype nor the haplotype observed in our patient cohort was identified in the Turkish Genome Project Portal participants. To further investigate the specificity of this haplotypic structure, the same SNP markers were evaluated in patients carrying other *CYP4V2* variants. Non-carriers did not demonstrate preservation of this extended nine-SNP haplotype block. Although a small subset of non-carriers of c.802–8_810del17insGC (4 of 26 patients) showed partial preservation of a shorter three-SNP block, the marker rs7862918 was not retained in these patients (Supplementary Fig. 1 ).

### Genotype-phenotype relationships in patients

The patients were stratified into three groups based on the number of *CYP4V2* alleles carrying loss-of-function (LOF) variants, including nonsense and splicing variants. Clinical characteristics were compared among patients with biallelic LOF variants (*n* = 11), monoallelic (*n* = 4), and those with no LOF variants (*n* = 17). There were no significant differences in AO, BCVA, BCVA change/year, CFT, SCT, MD, disease stage, or presence of ORT (*p* > 0.05, Supplementary Table 4). Notably, three patients with an AO under 12 years possessed biallelic LOF variants, while one patient with an AO over 35 also had biallelic LOF variants.

**Fig. 3:**
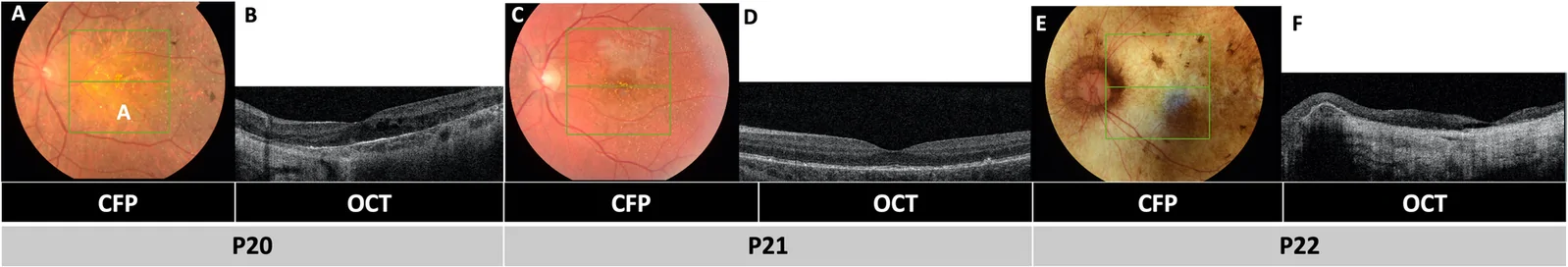
Color fundus photography (CFP) and optical coherence tomography (OCT) images of BCD patients with the homozygous variant c.802–8_810del17insGC. **A**: CFP image of the left eye of P20 showing stage 1 fundus findings with retinal crystals scattered throughout the posterior pole with some peripheral pigment clumping; **B**: OCT image of the left eye of P20 showing outer retinal tubulations (ORT) at stage 1; **C**: CFP image of the left eye of P21 showing stage 1 fundus findings with retinal crystals scattered throughout the posterior pole and the central RPE changes; **D**: OCT image of the left eye of P21 with hyperreflective crystals at RPE layer and without any ORT; **E**: CFP image of the left eye of P22 showing stage 3 fundus findings with severe widespread chorioretinal atrophy and pigment clumping areas; **F**: OCT image of the left eye of P22 showing severe retinal and choroidal thinning with the loss of ISOS band

Among six patients from four families with the homozygous c.802–8_810del17insGC variant, AO varied between 10 and 25 years. Two younger siblings (P20 and P21), aged 12 and 14, exhibited BCVA of 0.8 or better, with stage 1 fundus findings (Fig. 3). An older patient (P17) showed asymmetric BCVA, while the other two older siblings (P18, P19) presented with different severity despite similar ages. The oldest patient (P22) had stage 3 disease with severe findings, including widespread chorioretinal atrophy, the thinnest CMT and SCT (Fig. 3). Clinical characteristics were also compared among patients having the homozygous c.802–8_810del17insGC variant and the patients with other genotypes. There were no significant differences in AO, BCVA, BCVA change/year, CFT, SCT, MD, or disease stage (*p* > 0.05, Supplementary Table 5).

The novel homozygous missense variant, p.His155Pro, was found in P29, who remained asymptomatic and was incidentally diagnosed at age 22. At 32, he maintained a BCVA of 1.0 with minimal retinal findings (Fig. 1). P13 harboured a heterozygous novel splicing variant, c.802-1G > A, alongside a pathogenic missense variant (p.Ile111Thr) and had stage 3 findings at age 28. Siblings P25 and P26 both had the homozygous novel splicing variant c.802–2 A > C; the younger sister showed severe stage 3 findings, while the older brother had better BCVA and stage 2 findings (Fig. 1).

The study included six families with sibling pairs, revealing phenotypical variations—including AO, BCVA, and disease stage—among siblings of similar ages. Additionally, five unrelated patients harbouring the same *CYP4V2* variants exhibited differing disease severities. For instance, while P10 had stage 1 disease and a BCVA of 0.8, younger patients P11 and P12 exhibited worse visual acuity and more severe fundus findings (Table 1).

## Discussion

This study provided the genotype and ocular phenotype characteristics in 32 Turkish BCD patients. All patients had biallelic *CYP4V2* mutations. Twelve different variants were detected, three of which were novel. To our knowledge, this is the first report to present detailed genetic and clinical characteristics of Turkish BCD patients and represents one of the largest BCD cohorts described outside East Asian populations.

One of the most striking findings was the unexpectedly high allelic frequency of the c.802–8_810del17insGC variant in our cohort. This variant has been reported as the most common in East Asian BCD patients [10, 11, 30]. Jiao et al. suggested that this variant originated in the Chinese population and subsequently spread to Japan and Korea [11]. In the gnomAD v4.1.0 database, the reported allele frequency is 0.0028 in East Asian populations and 9.32 × 10⁻⁶ in the European (non-Finnish) population (11 alleles among 1,179,970 total), while the variant has not been detected in American, African, South Asian, or Middle Eastern populations (https://gnomad.broadinstitute.org/gene/ENSG00000145476, last accessed 30 November 2025). Moreover, this variant has previously been reported in a small number of BCD patients of non–East Asian ancestry, including four heterozygous individuals of Caucasian/European background and two homozygous individuals of European or “white other” origin [16, 20, 31]. In contrast, our study revealed an allelic frequency of 0.15 among unrelated Turkish BCD patients. Importantly, it was not identified in the Turkish Genome Project Portal, which includes genomic data from 557 healthy Turkish individuals. The haplotype findings provide further insight into the unexpectedly high frequency of c.802–8_810del17insGC in our Turkish cohort. Rather than representing a randomly recurring event, the conserved extended nine-SNP haplotype block in all individuals harboring the homozygous c.802–8_810del17insGC variant strongly suggests that this variant is carried on a shared ancestral chromosomal segment. The substantial overlap with previously reported East Asian founder haplotypes, together with divergence at specific flanking loci, is more compatible with a common founder origin followed by local chromosomal recombination than with multiple independent mutational events [11]. Particularly noteworthy is the divergence observed at rs7862918, while the core haplotypic region adjacent to the mutation remained preserved. This pattern may reflect historical recombination involving an ancestral mutant chromosome after its introduction into the local population. Likewise, the similarity of the terminal SNPs to the Korean haplotype suggests that the Turkish mutant chromosome shares partial similarity with more than one reported East Asian founder background, further supporting an ancestral migration and admixture model [11]. The comparison with patients carrying other *CYP4V2* variants further strengthens this interpretation. The absence of the extended conserved haplotype in non-carriers, together with the lack of rs7862918 retention even in the few patients showing partial three-SNP similarity, indicates that this extended haplotypic structure is unlikely to represent a common local background. Instead, it appears to be specifically linked to the c.802–8_810del17insGC mutant chromosome, supporting the possibility of a shared ancestral lineage. At the same time, these findings should be interpreted with appropriate caution. Because the available data are derived from targeted sequencing with limited SNP coverage, full phase-resolved haplotype reconstruction and formal ancestry inference are not possible. Therefore, our results should be interpreted as being consistent with, rather than definitive proof of, a founder mutation followed by recombination and admixture. From a broader population genetics perspective, Türkiye has long been a crossroads for migrations from Asia to Europe. Early genomic studies demonstrated that the Turkish population is nearly equidistant from European and Han Chinese populations [32]. More recent whole-genome sequencing studies revealed that Turkish genetic variation clusters primarily with South European populations but also carries signatures of East Asian ancestry [33]. A nationwide genomic study involving 3362 Turkish individuals confirmed that admixture, inbreeding, and migration events have shaped the modern Turkish genome, with Central Asian populations contributing approximately 9.6% [34]. These data may help explain the relatively high frequency of the c.802–8_810del17insGC mutation in our cohort, partially mirroring findings in East Asia. Consistent with this mixed genetic background, our cohort also included c.332T > C, the most common European BCD variant, in four unrelated patients [11]. In contrast, two other frequent variants in our population, c.1198 C > T and c.252 C > A, have not been reported as common in previously studied populations, further reflecting the unique population history of Türkiye at the intersection of European and Asian genetic influences [4, 10–21, 30].

In this cohort, we identified three novel *CYP4V2* variants: c.802–2 A > C and c.464 A > C were homozygous in patients and c.802-1G > A was found with another previously identified pathogenic variant in trans position, creating a biallelic effect on *CYP4V2*. All three were absent from GnomAD and Turkish Genome Project portals. c.802–2 A > C and c.802-1G > A both affect splicing and according to ACMG guidelines, those variants were judged as LP (PVS1_ Strong+PM2). c.464 A > C, p.His155Pro was a missense variant in exon 4 of the *CYP4V2*. This alteration results from an A to C substitution at nucleotide position 464, causing the histidine (H) at amino acid position 155 (in the P450 domain of the protein) to be replaced by a proline (P). Histidine is a medium-sized polar amino acid. However, proline is a medium-sized hydrophobic amino acid. The His155 residue was conserved across species with high conservation score (phyloP100: 8.806, Supplementary Fig.2), and multiple lines of computational evidence indicated a likely deleterious effect for the p.His155Pro variant. Therefore, this variant was also judged as LP according to ACMG guidelines (PM2, PM3_Supporting, PP3, PP4).

The homozygosity rate in our cohort was remarkably high (87.5%) which is partially consistent with the elevated parental consanguinity rate observed (43.7%) and with previously reported high rates of consanguineous marriages in Türkiye [35]. However, nearly half of the homozygous patients in our study denied parental consanguinity, suggesting that consanguinity alone cannot fully account for the high homozygosity. Furthermore, several unrelated patients were found to carry identical *CYP4V2* mutations. Recent population-genetic analyses in Turkish cohorts have demonstrated substantial autozygosity and long runs of homozygosity (ROH), indicating underlying population substructure and elevated background relatedness [34, 36]. These findings support the possibility that endogamous mating within regional, ethnic, or geographic subgroups — even in the absence of documented consanguinity — may contribute to increased autozygosity and the persistence of founder mutations. In our cohort, the c.802–8_810del17insGC variant showed partial haplotype sharing among apparently unrelated families, which is consistent with—but cannot conclusively demonstrate—a possible founder effect similar to that reported in East Asian populations.¹¹ For other recurrent *CYP4V2* mutations, regional founder effects remain a plausible hypothesis, though dedicated haplotype analyses will be required to confirm this. Previous reports from different geographical regions have reported various recurrent mutations, which seem to be related to geographic or ethnic backgrounds [4, 10–21, 30]. However, the reasons for these regional differences need to be further studied.

Genotype–phenotype associations in BCD is challenging because the clinical parameters used for comparison—such as BCVA, CFT, SCT, MD, and disease stage—are dynamic and change over the course of the disease, whereas genotype remains constant. As a result, cross-sectional assessments may be confounded by the timing of evaluation and interindividual variability in disease progression. Among the variables analyzed in this study, age of onset represents the most stable parameter, and although differences did not reach statistical significance, biallelic loss-of-function variants showed a tendency toward earlier onset. To minimize the influence of follow-up duration and temporal variation in disease severity, we additionally examined annual BCVA change as a functional progression metric. Incorporating this parameter allowed a more accurate comparison between genotype groups, although no statistically significant differences were observed, likely in part due to the relatively small sample size. However, previous studies have also reported inconsistent findings regarding such correlations.¹ Notably, for one of the most common variants in East Asia, c.802–8_810del17insGC, large cohort studies have reported conflicting findings regarding its association with disease severity compared with other genotypes [1, 10, 20, 30, 37, 38]. In a recent study, Murakami et al. demonstrated that BCD patients carrying this deleterious variant did not exhibit a more severe phenotype, including no faster rate of vision loss, than patients with other genotypes.³^8^ Similarly, in our cohort, individuals with this variant did not show a more severe clinical phenotype compared with those harbouring other *CYP4V2* mutations (Supplementary Table 5).

In our cohort, considerable variability was observed even among patients with identical variants, both between families and within families. For example, two unrelated patients homozygous for p.Ile111Thr showed markedly different disease severity and AO (P10 and P12), while two siblings with the c.802–2 A > C variant also displayed striking phenotypic differences (P25 and P26). These findings reinforce previous reports that BCD demonstrates wide clinical heterogeneity with limited genotype–phenotype correlation [1, 4, 10–21, 30]. This variability may be influenced by additional genetic modifiers or environmental factors such as diet, which could affect lipid metabolism and disease expression [9].

Ocular symmetry was also evaluated in our cohort. Although previous studies generally described symmetrical involvement, Han et al. reported strong interocular symmetry in visual field and ERG, but weaker symmetry in BCVA [39]. Consistently, we observed interocular asymmetry in BCVA (7/32 patients) and OCT thickness (2/32 patients), but not in visual field MD values. This supports the view that visual acuity may be more asymmetric than visual field in BCD and other inherited retinal dystrophies such as choroideremia and retinitis pigmentosa [40–43].

OCT remains a crucial tool in the evaluation of BCD, as it can detect early RPE and choroidal atrophy not visible on fundus examination. Moreover, ORT, a sign of active photoreceptor degeneration, can be detected using OCT. In our study, we detected ORT in all stages, affecting a total of 40.6% of patients. In literature, ORTs were mostly reported in stage 2 and 3 [13, 23, 44]. However, Wang et al. presented the incidence of ORT as 50.0% (7/14 eyes) in stage 1, 92.1% (35/38 eyes) in stage 2, and 35.7% (10/28 eyes) in stage 3 and suggested that the presence ORT was a sign of onset of RPE atrophy in early stage and slower progression in advanced stages [45]. We did not conduct a progression analysis; however, we had two sibling groups supporting this suggestion. P20 and P21 both had stage 1 BCD. BCVA, CMT, and SCT were higher in P21, who had no sign of ORT in OCT. P23 and P24 both had stage 3 BCD. BCVA, CMT, and SCT were higher in P23, who had ORTs detected in OCT (Table 1). Serial measurements of CMT and SCT in BCD patients are also used to monitor the disease progression [14, 23]. All of our patients had measured CMT and SCT values. Despite the lack of statistically significant difference between mean CMT and SCT values of BCD patients when grouped according to the genotype, the correlation of BCVA with CMT and SCT was positive and significant (*p* < 0.001, Supplementary Table 2) in our study consistent with previous studies [23, 46].

In our study, foveoschisis was the most detected posterior segment complication in BCD patients (4/32). CME was detected only in 2 patients, and CNV was not shown in any BCD patients. Foveoschisis was detected in stage 3 patients of our cohort; however, CME was seen in stage 1 and 2 patients. Posterior segment complications were reported as uncommon in BCD, and the underlying pathogenesis of these complications has remained unclear [1, 2]. Therefore, we had no clinical or genetic clues to predict patients at risk for these complications.

This study has several limitations. First, it was retrospective in design, and analyses were restricted to available clinical data. Second, not all patients underwent electrophysiological testing, which limited the statistical assessment of the relationship between BCVA and mfERG amplitudes. Third, genotype–phenotype correlations did not reach statistical significance, likely due to the relatively small sample size.

In conclusion, this study presents the first detailed analysis of genotype–phenotype characteristics in Turkish BCD patients and represents one of the largest cohorts reported from Europe. In 26 probands, we identified 12 different *CYP4V2* variants, including three novel variants, thereby expanding the known mutational spectrum of BCD. Our findings highlight marked intra- and interfamilial phenotypic variability and confirm the absence of clear genotype–phenotype correlations. As an unexpected finding, c.802–8_810del17insGC, which is mainly seen in East Asian populations, was found to be one of the common variants in the Turkish BCD population. Additionally, a common European variant, c.332T > C, was also prevalent in our cohort, supporting the contribution of both Asian and European ancestries to the genetic structure of the Turkish population.

## Supplementary Information

Below is the link to the electronic supplementary material.

Supplementary Material 1 (DOCX 19.6 KB)

Supplementary Material 2 (DOCX 17.3 KB)

Supplementary Material 3 (DOCX 10.6 KB)

Supplementary Material 4 (DOCX 18.3 KB)

Supplementary Material 5 (DOCX 19.3 KB)

Supplementary Material 6 (DOCX 760 KB)

## Data Availability

The data that support the findings of this study are available from the corresponding author upon reasonable request.
