# Supplementary material for: Bietti crystalline dystrophy in Türkiye: A genetic crossroads between Asia and Europe
Source: Graefes Arch Clin Exp Ophthalmol. 2026 Apr 20;264(10):3181–92. doi: 10.1007/s00417-026-07245-w (PMC13635032; doi:10.1007/s00417-026-07245-w)
Supplement: Supplementary file 1 [file 417_2026_7245_MOESM2_ESM.docx]

Supplementary Table 2. Correlation analysis between BCVA, CMT, SCT, AO and MD values of BCD patients

|  | | BCVA | CMT | SCT | MD | AO |
| --- | --- | --- | --- | --- | --- | --- |
| BCVA | Pearson r | 1 | 0.662^**^ | 0.793^**^ | -0.691^**^ | 0.007 |
|  | p |  | 0,000 | 0.000 | 0,000 | 0.971 |
| CMT | Pearson r | 0.662^**^ | 1 | 0.628^**^ | -0.413^*^ | -0.198 |
|  | p | 0.000 |  | 0.000 | 0.019 | 0.278 |
| SCT | Pearson r | 0.793^**^ | 0.628^**^ | 1 | -0.708^**^ | -0.222 |
|  | p | 0.000 | 0.000 |  | 0.000 | 0.222 |
| MD | Pearson r | -0.691^**^ | -0.413^*^ | -0.708^**^ | 1 | 0.149 |
|  | p | 0.000 | 0.019 | 0.000 |  | 0.416 |
| AO | Pearson r | 0.007 | -0.198 | -0.222 | 0.149 | 1 |
|  | p | 0.971 | 0.278 | 0.222 | 0.416 |  |

CMT: Central Macular Thickness, SCT: Subfoveal Choroidal Thickness, MD: Mean Deviation BCVA: Best Corrected Visual Acuity, AO: Age of onset

For the BCVA, CMT and SCT values worse eye was used for statistical analysis.

**. Correlation is significant at the 0.01 level.

*. Correlation is significant at the 0.05 level.
