# Supplementary material for: Bietti crystalline dystrophy in Türkiye: A genetic crossroads between Asia and Europe
Source: Graefes Arch Clin Exp Ophthalmol. 2026 Apr 20;264(10):3181–92. doi: 10.1007/s00417-026-07245-w (PMC13635032; doi:10.1007/s00417-026-07245-w)
Supplement: Supplementary file 2 [file 417_2026_7245_MOESM4_ESM.docx]

**Supplementary Table 4.** Comparison between genotypes and clinical findings

|  | | **Genotype** | |  |
| --- | --- | --- | --- | --- |
|  | | LOF=0/1  (Mean/std)  n=21 | LOF=2  (Mean/std)  n=11 | p value |
| AO | | 22.7/7.2 | 20.4/9.3 | 0.481 |
| BCVA | | 0.2/0.3 | 0.3/0.4 | 0.558 |
| BCVA change/year*  (LogMAR) | | 0.094/0.121 | 0.024/0.038 | 0.075 |
| CMT | | 106.5/61.6 | 138.0/64.8 | 0.223 |
| SCT | | 85.6/57.8 | 131.1/109.4 | 0.785 |
| MD | | 27.7/6.7 | 25.55/10.3 | 0.796 |
| Stage | 1 | 2 | 2 | 0.413 |
|  | 2 | 8 | 4 |  |
|  | 3 | 11 | 5 |  |
| ORT | present | 13 | 6 | 0.721 |
|  | absent | 8 | 5 |  |

CMT: Central Macular Thickness, SCT: Subfoveal Choroidal Thickness, MD: Mean Deviation BCVA: Best Corrected Visual Acuity, AO: Age of onset, LOF: Loss of function variant, std: standard deviation, ORT: Outer retinal tubulation

For the BCVA, BCVA change, CMT, SCT and MD values worse eye was used for statistical analysis.

* For BCVA change/year, LOF=0/1 group included 20 patients and LOF=2 group included 9 patients due to nonavailable data.

For the comparison of AO, BCVA, BCVA change/year, CMT, SCT, and MD values between genotype groups Mann-Whitney test was used; for the comparison of stage and ORT between genotype groups Chi-square test and Fisher's exact test was used respectively.
