# Supplementary material for: Bietti crystalline dystrophy in Türkiye: A genetic crossroads between Asia and Europe
Source: Graefes Arch Clin Exp Ophthalmol. 2026 Apr 20;264(10):3181–92. doi: 10.1007/s00417-026-07245-w (PMC13635032; doi:10.1007/s00417-026-07245-w)
Supplement: Supplementary file 3 [file 417_2026_7245_MOESM5_ESM.docx]

**Supplementary Table 5.** Comparison between genotypes and clinical findings

|  | | **Genotype** | |  |
| --- | --- | --- | --- | --- |
|  | | Patients with  other genotypes  (Mean±std)  n=26 | Patients with c.802-8_810del17insGC allele  (Mean±std)  n=6 | p value |
| AO | | 23.0 ± 7.95 | 17.0 ± 6.03 | 0.113 |
| BCVA | | 0.232 ± 0.303 | 0.310 ± 0.461 | 0.697 |
| BCVA change/year  (LogMAR) | | 0.062 ± 0.075 | 0.010 ± 0.022 | 0.090 |
| CMT | | 117.2 ± 60.2 | 118.0 ± 82.9 | 0.961 |
| SCT | | 96.6 ± 68.5 | 121.3 ± 127.3 | 0.961 |
| MD | | 27.31 ± 7.33 | 25.58 ± 11.40 | 0.923 |
| Stage | 1 | 3 | 2 | 0.340 |
|  | 2 | 10 | 1 |  |
|  | 3 | 13 | 3 |  |

CMT: Central Macular Thickness, SCT: Subfoveal Choroidal Thickness, MD: Mean Deviation BCVA: Best Corrected Visual Acuity, AO: Age of onset, LOF: Loss of function variant, std: standard deviation, ORT: Outer retinal tubulation

For the BCVA, BCVA change, CMT, SCT and MD values worse eye was used for statistical analysis.

For the comparison of AO, BCVA, BCVA change/year, CMT, SCT, and MD values between genotype groups Mann-Whitney test was used; for the comparison of stage between genotype groups Chi-square test was used.
