# Supplementary material for: Bietti crystalline dystrophy in Türkiye: A genetic crossroads between Asia and Europe
Source: Graefes Arch Clin Exp Ophthalmol. 2026 Apr 20;264(10):3181–92. doi: 10.1007/s00417-026-07245-w (PMC13635032; doi:10.1007/s00417-026-07245-w)
Supplement: Supplementary file 4 [file 417_2026_7245_MOESM6_ESM.docx]

**Supplementary Figure 1:** Schematic representation of the *CYP4V2* haplotype structures surrounding the pathogenic variant c.802-8_810del17insGC in previously reported East Asian populations (Jiao et al.)^11^, in our study cohort with and without the c.802-8_810del17insGC variant, and in the Turkish Genome Project Portal. Conserved allele blocks are shaded in green. The pink-shaded SNP (rs7862918, C>T) was observed only in our study cohort. *indicates the number of alleles carrying the shown haplotype / total number of alleles harboring the pathogenic variant in the respective populations. ** indicates the number of patients carrying other CYP4V2 variants / total number of BCD patients in our study cohort.

NA, genotype data not available.

**Supplementary Figure 2:** The alignment of novel missense mutation of *CYP4V2* gene, c.464A>C, p.His155Pro, with the corresponding segments in seven species. Amino acid marked with yellow in the column are highly conserved among all shown species in H155P.
